# Prevalence and associated factors of Carpal Tunnel Syndrome (CTS) among medical laboratory staff at King Saud University Hospitals, KSA

**DOI:** 10.12669/pjms.312.6831

**Published:** 2015

**Authors:** Shaffi Ahamed S, Bardeesi Anas M, Altwair Aref A, AlMubarak Abdulrahman A

**Affiliations:** 1 Shaffi Ahamed S, Ph.D, Department of Family and Community Medicine, College of Medicine, King Saud University, Riyadh, Saudi Arabia; 2 Bardeesi Anas M, MBBS, Department of Family and Community Medicine, College of Medicine, King Saud University, Riyadh, Saudi Arabia; 3 Altwair Aref A, MBBS, Department of Family and Community Medicine, College of Medicine, King Saud University, Riyadh, Saudi Arabia; 4 AlMubarak Abdulrahman A, MBBS. Department of Family and Community Medicine, College of Medicine, King Saud University, Riyadh, Saudi Arabia

**Keywords:** Carpal tunnel syndrome, laboratory workers, risk variables, Boston Carpal Tunnel Questionnaire

## Abstract

**Background and Objectives:**

Carpal tunnel syndrome (CTS) is a group of symptoms resulting from local compression of the median nerve at the wrist leading to its subsequent functional impairment and local ischemia of the nerve. Our objective was to determine the prevalence and commonly reported symptoms of CTS in the laboratory workers of King Saud University (KSU) hospitals and to identify the associated variables with CTS.

**Methods:**

This was a quantitative observational cross-sectional study which was conducted in KSU hospitals’ laboratories with a total of 225 participants by using a standardized questionnaire known as “ Boston Carpal Tunnel Questionnaire (BCTQ). Data Analysis was carried out by IBM SPSS Statistics software version 21.0.

**Results:**

Out of the 225 participants, 57 were found to be severely symptomatic with a prevalence of 25.3%. Among the severely affected participants, females were more than males (58% > 42%) and the difference was statistically significant (p=0.045). Technicians affected (91.2%) were more than attendants (8.8%) and the difference was statistically significant (p=0.042).

**Conclusion:**

The prevalence of Carpal tunnel syndrome in KSU hospitals’ medical laboratory staff (25.3%) was close to what was found in literature (21.5%). So laboratory workers are at risk of developing CTS, especially females and technicians with the dominant hand most likely to be affected.

## INTRODUCTION

Carpal tunnel syndrome (CTS) is a group of symptoms resulting from the local compression of the median nerve at the wrist which results in a subsequent functional impairment and local ischemia of the nerve within the tunnel. The carpal tunnel is a narrow passageway that is located on the palmer or anterior aspect of the wrist bounded by bones and ligaments. Compression of the nerve results in symptoms that characterize the syndrome, including numbness, pain and, eventually, hand weakness.^1^,^2^

Laboratory occupations fall under the category of occupational risks of Carpal Tunnel Syndrome due to the performance of complex work that requires repetitive and accurate hand movement.^3^-^5^ Most of laboratory workers are actively involved in tasks requiring use of both hands for 4-6 hours per day.^6^ Although earlier studies on laboratory workers have tackled the risk factors for the development of CTS and measures that can be used to prevent it, there are no studies reporting the prevalence of CTS in laboratory workers. Due to shortage of local studies that prove this finding, we wish to compare the local and international data after completion of the study. We hope our study would educate the public in general, and the laboratory workers specifically about the risk they have to develop Carpal Tunnel Syndrome and what risk factors are involved in the development of the syndrome. The aims of this present study were to determine the prevalence of CTS among laboratory workers at KSU hospitals. We also intend to determine the associated factors and symptoms associated with developing CTS in laboratory workers at KSU hospitals.

## METHODS

A quantitative observational cross-sectional study was conducted among the Medical laboratories staff of King Saud University Hospitals (King Khalid University hospital and King Abdulaziz University Hospital) in Riyadh, Kingdom of Saudi Arabia over the period from 1st of September 2013 until the 1st of April 2014. The laboratory units in King Abdulaziz University Hospital include Phlebotomy, Clinical Biochemistry, Histology and Cytology, Immunology, Hematology, Bacteriology and Virology. The laboratory units in King Khalid University Hospital include Phlebotomy, Clinical Biochemistry, Histology, Cytology, Immunology, Hematology, Bacteriology, Virology, Cytogenetics, Molecular Biology, Parasitology, Mycology and Electron microscopy. Assuming a prevalence of 21.5%,^7^ with a precision of within 6% and at, *Z*_a/2_= 1.96, and anticipating 40% non-response, a total of 252 participants constitute the sample size of this study. As the target population is limited (274 laboratory staff), consent was obtained from all the staff to participate in the study. “The Boston Questionnaire” developed by Levine-Katz for the assessment of symptoms severity and functional status in carpal tunnel syndrome^8^ was used to collect the data. It is a self-administered questionnaire that consists of two sections, with a total of 19 questions. There are five stems for each question, adopting a ‘stem score’ from 1(mild) to 5(severe). Questions concerning the first section, entitled as symptoms severity scale, are 11 questions checking for: pain severity during day and night times, time of pain during the day, weakness, tingling sensation felt at night, how frequently did that tingling occur and whether there is numbness or not. For each question, five possible answers are there numbered from 1 to 5, arranged in an ascending order of symptoms severity. Therefore, 1 correlates to asymptomatic, 2 to mild symptoms, 3 to moderate symptoms, 4 to intense symptoms and 5 correlates to severe symptoms. For the second section - entitled as functional status scale- eight questions are there, where each one refers to a functional activity. The listed activities are “ writing, buttoning clothes, ability to hold a book while reading, holding a telephone hang, housekeeping, opening a glass vial cap, carrying market bags, bathing and dressing.” Each activity has five difficulty degrees, where degree 1 means no difficulty, degree 2 little difficulty, degree 3 moderate difficulty, degree 4 intense difficulty and degree 5 cannot perform the activity at all due to hands and wrists symptoms. All of the answers should refer to the symptoms which were experienced in a two-week time period, because we are trying to be as accurate as possible. The questionnaire was also translated properly into Arabic language and was revised to clear any ambiguity, and so it was used together with our modified English version to include non-Arabic speakers. The questionnaire also includes the variables: age, gender, years of occupation, BMI and work pattern that are all related to Carpal Tunnel Syndrome development. Toward ethical considerations, a consent to participate in the study was obtained from all the study subjects. No incentives or rewards were given to participants. Ethical approval was obtained from the Institutional review board of the hospital.

### Data analysis

Data were entered and analyzed using the IBM SPSS Statistics software (version 21). The study subjects were grouped according to their age, gender, years of occupation, occupation, BMI, and symptomatic hand. Age groups were as follows; ≤ 30 years, 31-45 years, and > 45 years. Years of occupation were grouped as follows; < 5 years, 5-10 years, and > 10 years. Occupation were categorized into technician and assistant. Body Mass Index (BMI) was categorized into normal (18-24), overweight (25-30), and obese (>30). Symptomatic hand was grouped to right, left, both, and asymptomatic. The outcome variable was categorized using percentiles to Mild, Moderate, and Severe in the Symptom Severity Scale (SSS) and to Mild-moderate and Severe in the Functional Status Scale (FSS). The three categories (mild, moderate, and severe) of susceptibility to having CTS symptoms were based on the SSS. Individuals suspected of having mild CTS symptoms are within the 50th percentile. Individuals suspected of having moderate CTS symptoms are within the 50^th^-75^th^ percentile. Individuals suspected of having severe CTS symptoms are above the 75^th^ percentile. The two categories (mild-moderate and severe) of susceptibility to having CTS symptoms will be based on the FSS. Individuals suspected of having Mild-moderate CTS symptoms will be within the 75^th^ percentile. Individuals suspected of having severe will be within the 4^th^ quartile (above 75^th^ percentile). Karl Pearson’s Chi-square test was used to observe the association between categorical study and outcome variables. Student’s t-test for independent samples and one-way analysis of variance was used to compare the mean values of the total scores of both SSS and FSS in relation to each of the associated categorical variables.

## RESULTS

Of the 225 study subjects, there were 122(54.2%) male subjects and 104(46.2%) and with age <= 30 years. About 104(46.2%) were with < 5 years of occupation and 191(84.9%) of them were technicians. Normal BMI was observed in 101(44.9%) subjects. Symptomatic hand as right hand was reported by 76(33.8%) of study subjects.(Table-I) By using symptom severity scale it was found that about 108(48%) study subjects had mild symptoms, 60(26.7%) were with moderate symptoms and 57(25.3%) were with severe symptoms. Among those 57 subjects, the order of the most frequently reported symptoms were as follows: pain (82.5%), weakness (73.7%), tingling (70.2%), nocturnal exacerbations (66.7%), numbness (61.4%) and difficulty grasping (31.6%). By using Functional severity scale it was observed that 155 (68.9%) were with moderate symptoms and 70(31.1) with severe symptoms (Table-II).

**Table-I:** Socio-Demographic Characteristics of Study Subjects.

Characteristics	Frequencies (%)
*Age*	
≤ 30	104 (46.2)
31 – 45	75 (33.3)
> 45	46 (20.4)
*Gender*	
Male	122 (54.2)
Female	103 (45.8)
*Years of occupation*	
< 5 years	104 (46.2)
5-10 years	44 (19.6)
>10 years	77 (34.2)
*Type of occupation*	
Technician	191 (84.9)
Assistant	34 (15.1)
*BMI*	
Normal	101 (44.9)
Overweight	83 (36.9)
Obese	41 (18.2)
*Symptomatic hand*	
Right	76 (33.8)
Left	29 (12.9)
Non-symptomatic	108 (48)
Both	12 (5.3)

**Table-II:** Distribution of Symptom Severity Scale and Functional Severity Scale Categories.

Scale	SSS (%)	FSS (%)
Mild	108 (48.0)	---
Moderate	60 (26.7)	155 (68.9)
Severe	57 (25.3)	70 (31.1)

The comparison of mean values of the SSS and FSS scores shows statistically significant difference in the mean values of SSS in relation to gender and occupation. That is the mean SSS score is significantly higher in female study subjects when compared with male subjects (t=-2.60; p=0.01) and the mean SSS score is significantly higher in technicians when compared with assistants in laboratory(t=2.08; p=0.04). Similarly the mean FSS score was statistically significantly higher in technicians when compared with assistants working in laboratory (t=4.25; p<0.0001). The mean values of SSS and FSS scores are not statistically significantly different across the three age groups (<=30, 31-45, & >45 years), three categories of years of occupation(<5, 5-10 & > 10 years) and three categories of BMI (normal, overweight & obese). (Table-III) There is a highly statistically significant association between the study subjects dominant hands and their symptomatic hand in which, out of 94 right-handed participants with symptoms, 75 (79.8%) reported their right hands as being the symptomatic one and among the 11 participants who were left-handed with symptoms, 10 (90.9%) of them reported their left hand as the one being symptomatic(χ^2^= 30.96; p<0.0001).(Table-IV)

**Table-III:** Comparison of mean values of SSS and FSS in relation to risk variables.

	SSS mean (sd.,)	t-value/ F-value	p-value	FSS mean (sd.,)	t-value/ F-value	p-value
*Gender*						
Male	13.78(4.23)	-2.60	0.01*	9.14(2.26)	-1.85	0.067
Female	15.62(6.07)			9.94(3.89)		
*Type of occupation*						
Technician	14.90(5.30)	2.08	0.042*	9.70(3.34)	4.25	0.0001*
Assistant	13.09(4.54)			8.44(0.99)		
Age						
≤ 30	14.1(4.33)	0.98	0.378	9.42(2.47)	0.08	0.923
31 – 45	14.97(6.39)			9.61(4.19)		
> 45	15.22(4.96)			9.52(2.43)		
*Years of occupation*						
< 5 years	13.85(4.75)	2.66	0.072	9.44(3.14)	0.41	0.664
5-10 years	15.91(5.57)			9.89(3.69)		
>10 years	14.94(5.51)			9.38(2.78)		
*BMI*						
Normal	13.93(4.26)	1.78	0.171	9(1.87)	2.43	0.090
Overweight	15(5.68)			9.9(3.81)		
Obese	15.56(6.22)			9.95(3.91)		

**Table-IV:** Distribution of Reported Symptomatic Hand in Relation to Dominant Hand.

Symptomatic hand (no. & %)			
	Right	Left	χ^2^-value	p-value
*Dominant hand*				
Right	75 (79.8)	19 (20.2)	30.9	<0.0001
Left	1(9.1)	10 (90.9)		

## DISCUSSION

This study with 225 study subjects, had identified 57 were having severe symptoms of CTS on Symptom Severity Scale and they were also found to be having deterioration in their Functional Status Scale as well. Thirteen participants have shown deterioration in their Functional Status Scale, but without expressing severe symptoms on the Symptom Severity Scale. Our interpretation of such finding gave two possibilities. Either they were having other unidentified musculoskeletal or neurological problem- not related to CTS- that affected their daily functions, or they were biased when answering the functional status scale section of the questionnaire.

In the study that was conducted to determine the prevalence of CTS in the medical college of Belgaum, India by Kamaraddi SV et al.^7^ 21.5% was the prevalence they got. Whereas the prevalence of CTS in our study is 25.3%. Although this prevalence is very close to others provided in the literature, our assessment is slightly different as we only identified severely symptomatic lab workers and couldn’t pronounce them as CTS cases. In comparison with other publications that studied the prevalence of CTS in other vocational groups at risk, variations in the prevalence rates were present. Among fish processing workers, CTS prevalence was reported at 15%, dental hygienists at 8.4%, dentists at 4.8%, construction workers at 8.2% and ski manufacturing workers at 15.4.^9^

CTS is well known to result in a group of symptoms frequently reported by patients. Those include: pain, numbness, tingling, thenar muscle wasting, weakness, nocturnal exacerbations and difficulty grasping items. We identified the most frequently reported symptoms in those being severely symptomatic. Upon analysis, the symptoms were in the following order: hand/wrist pain in 47 (82.5%), hand weakness in 42 (73.7%) tingling in 40 (70.2%), nocturnal exacerbations in 38 (66.7%), numbness in 35 (61.4%) and difficulty grasping in 18 (31.6%). When we compare these findings to those presented by Kamaraddi SV et al.^7^, we find that they reported the same symptoms but with lesser percentages. Numbness was reported in 6 (30%), hand/wrist pain in 18 (90%), tingling in 2 (10%), nocturnal exacerbations in 2 (10%), hand weakness in 4 (20%), difficulty in grasping of items in 1 (10%), plus two extra symptoms; thenar muscle wasting and motor weakness in 1 (5%). We found out that among 57 participants with severe symptoms of CTS, 30 (52.6%) had symptoms of CTS only in the right hand, 16 (28.1%) had symptoms in the left hand, and 11 (19.3%) had symptoms in their both hands.

There are certain conditions and occupations that predispose to CTS. Many and countless risk factors had been reported regarding this condition.^10^-^12^ The risk factors that were studied in the present study included age, gender, occupation, years of occupation, BMI and dominant hand. Of these, the statistically significant associated variables were gender, occupation, and dominant hand. Females were more than males in terms of severity of symptoms with a count of 33 compared to 24 males. Technicians outnumbered assistants in terms of severity of symptoms with a number of 52 in comparison with assistants who counted 5. This can be interpreted by the work pattern of technicians who actually deal more with laboratory instruments than assistants. Coming to hand dominance, 36.4% of right handed study subjects reported symptoms in their right hands and 52% of left handed study subjects reported symptoms in their left hands. This variation (percentage being higher in left handed lab workers) is due to the scarcity of left handed individuals. This suggests that there is an association between hand dominance and susceptibility of CTS development in that hand. Kamaraddi SV et al.^7^, found that age, years of occupation, and work pattern are statistically significant risk factors. Therefore, only occupation (work pattern) was a shared significant risk in both studies. So from the list of factors we suggested it would have a great influence on the severity of symptoms, age, years of occupation, and BMI had no statistically significant evidence relating them to the development of CTS.

This is a questionnaire based study and cases of CTS could not objectively be identified with the gold standard diagnostic tool. We were only able to refer to those severely symptomatic and highly suggestive of having or developing the syndrome, by using the total scores of SSS and FSS, which were calculated based on the responses of study subjects. Hence the prevalence of CTS we are reporting may be under or over estimated.

### Limitation

Although the Boston Questionnaire for CTS was originally designed to assess post-surgical outcomes in CTS patients, where the scores of SSS and FFS were compared between pre and post surgery of patients. In our study, this validated Questionnaire was used to determine the prevalence of CTS among the subjects who are at risk of developing CTS. Our methodology is similar to the study done by Kamaraddi SV et al^7^ where the prevalence of CTS was also determined by the Boston Questionnaire. So we believe using the validated Boston Questionnaire was better rather than using a self-developed tool which requires validation.

## CONCLUSION

We conclude that the prevalence of severe symptoms of CTS in the laboratory workers is higher in the technicians, when the diagnosis is made using symptom severity scale. Females have higher risk of developing CTS than males, and the lab worker’s dominant hand is the most likely one to be affected if the syndrome is to develop.
